# Sampling *Daphnia*'s expressed genes: preservation, expansion and invention of crustacean genes with reference to insect genomes

**DOI:** 10.1186/1471-2164-8-217

**Published:** 2007-07-06

**Authors:** John K Colbourne, Brian D Eads, Joseph Shaw, Elizabeth Bohuski, Darren J Bauer, Justen Andrews

**Affiliations:** 1 The Center for Genomics and Bioinformatics, and Department of Biology, Indiana University, Bloomington, Indiana 47405, USA; 2 Department of Biology, Dartmouth College, Hanover, New Hampshire 03755, USA; 3 Hubbard Center for Genome Studies, University of New Hampshire, Durham, New Hampshire 03824, USA

## Abstract

**Background:**

Functional and comparative studies of insect genomes have shed light on the complement of genes, which in part, account for shared morphologies, developmental programs and life-histories. Contrasting the gene inventories of insects to those of the nematodes provides insight into the genomic changes responsible for their diversification. However, nematodes have weak relationships to insects, as each belongs to separate animal phyla. A better outgroup to distinguish lineage specific novelties would include other members of Arthropoda. For example, crustaceans are close allies to the insects (together forming Pancrustacea) and their fascinating aquatic lifestyle provides an important comparison for understanding the genetic basis of adaptations to life on land versus life in water.

**Results:**

This study reports on the first characterization of cDNA libraries and sequences for the model crustacean *Daphnia pulex*. We analyzed 1,546 ESTs of which 1,414 represent approximately 787 nuclear genes, by measuring their sequence similarities with insect and nematode proteomes. The provisional annotation of genes is supported by expression data from microarray studies described in companion papers. Loci expected to be shared between crustaceans and insects because of their mutual biological features are identified, including genes for reproduction, regulation and cellular processes. We identify genes that are likely derived within Pancrustacea or lost within the nematodes. Moreover, lineage specific gene family expansions are identified, which suggest certain biological demands associated with their ecological setting. In particular, up to seven distinct ferritin loci are found in *Daphnia *compared to three in most insects. Finally, a substantial fraction of the sampled gene transcripts shares no sequence similarity with those from other arthropods. Genes functioning during development and reproduction are comparatively well conserved between crustaceans and insects. By contrast, genes that were responsive to environmental conditions (metal stress) and not sex-biased included the greatest proportion of genes with no matches to insect proteomes.

**Conclusion:**

This study along with associated microarray experiments are the initial steps in a coordinated effort by the *Daphnia *Genomics Consortium to build the necessary genomic platform needed to discover genes that account for the phenotypic diversity within the genus and to gain new insights into crustacean biology. This effort will soon include the first crustacean genome sequence.

## Background

Among the major groups of the phylum Arthropoda – Chelicerata, Myriapoda, Crustacea, Hexapoda (insects and relatives) – the crustaceans and insects are allies [1]. They are together classified as members of the Pancrustacea, although their reciprocal monophyly is currently disputed [2-4]. Despite this phylogenetic uncertainty for taxa that have likely diverged some 600 million years ago [5] the model crustacean *Daphnia *is expected to share genes that are central to arthropod biology and development with well studied insects, such as *Drosophila*, *Anopheles*, *Bombyx *and *Apis*. Indeed, gene-by-gene investigations have already demonstrated the functional conservation of selected loci involved in germline formation and embryonic patterning between representative crustaceans and insects [6-8]. Yet, these two classes of animals have also evolved in radically different environments; branchiopod crustaceans are adapted to aquatic habitats, while the insects are predominantly adapted to terrestrial habitats. It is therefore expected that proteins required for life in these particular environments will reflect the biotic and abiotic challenges faced by these particular taxonomic groups. Furthermore, model crustaceans like *Daphnia *(order Cladocera) have a highly specialized mode of reproduction called cyclical parthenogenesis [9], which involves environmental sex determination and is derived from obligate sex [10]. Thus, the genetic control of cyclical parthenogenesis may have arisen from modifications in the structure and/or the regulation of arthropod reproductive genes. Similar mechanisms may apply for a variety of other adaptations, including *Daphnia*'s morphological transmutations in response to predator kairmones (called cyclomorphosis), their ability to shift from direct development into diapause within ephemeral habitats, and mechanisms for acclimating to both natural and anthropogenic stressors such as hypoxia or metal contamination. The evolution of these traits is expected to involve species-specific modifications of gene regulation, the restructuring of genes common to arthropods [11] and innovations unique to their aquatic habitats. Additionally, transitions in breeding systems and the origins of other adaptive traits probably also involve novel genes or lineage specific gene family expansions [12].

Comparative studies into the functional conservation of genes and the genetic basis of adaptation are made easier by the rapid development of genomic data and technologies. For example, cross-species comparisons within the Nematoda, based on over 265,000 expressed sequence tags (ESTs) from 30 species, indicate that roughly 40% of the 93,000 characterized genes have no known homologues within the phylum [13] while 23% of genes are unique to each species [14]. These large differences in gene content reflect (in part) the ecological diversity of the sampled nematodes, including free-living species and others that are plant or animal parasites. Not surprising, genetic novelty can be linked to an organism's specialized lifestyle. For instance, unique sequences of the parasitoid nematode *Nippostrongylus brasiliensis *are nearly 10 times enriched with signal peptides compared to conserved sequences, suggesting that the proliferation of these genes is accelerated because of their defensive role against host immunity [15]. Aside from the deeply divergent nematode comparisons [16], studies have thus far been restricted to the eukaryotic crown group [17], contrasts among species from the same order (i.e., primates, rodents) or species belonging to a similar class (insects). This situation is a consequence of the currently sparse coverage of genome sequencing projects along the metazoan phylogenic tree. Therefore, the addition of a crustacean to the growing list of sequenced insect genomes will expand the analysis of gene content among the ecologically diverse arthropod assemblage and provide information on the degree of protein family expansions by appropriately rooting the insect phylogeny.

Given the diversity of crustacean body plans, their fascinating biology and their key phylogenetic relationship to model invertebrates with sequenced genomes, the paucity of crustacean molecular data is striking. Indeed, protein sequences from *all *crustaceans represent only 0.1% of 6.9 million records in the NCBI taxonomic database. Among crustaceans, the freshwater zooplankton *Daphnia pulex *has a rich history of attracting attention from biologists – which now involves researchers in the fields of ecology and evolution, development, toxicology and genetics. Here, we present the first systematic study of transcribed sequences in *D. pulex*. The results of our survey highlight the diversity of crustacean genes that are shared with insects, and also uncovers gene family expansions that likely reflect the demands of aquatic existence, particularly homeostasis, defense/immunity, oxyregulation, and chemical sensing. In companion papers, we describe the development of the first *D. pulex *microarray used to investigate sex-biased transcriptional regulation of these genes (Eads et al. submitted) and the genomic response of this sentinel species to toxic metals commonly found in the environment (Shaw et al. submitted, and in prep). These studies are the initial steps in a coordinated effort by the *Daphnia *Genomics Consortium [18] to build the necessary data banks and reagents needed to discover genomic changes responsible for the phenotypic diversity within the genus and to gain new insights into crustacean biology. This effort will soon include the first crustacean genome sequence.

We report on the construction of *D. pulex *cDNA libraries and the sequences and analyses of 1,546 ESTs, of which 1,414 represent approximately 787 nuclear genes. We analyze these transcribed sequences against those of sequenced model invertebrates. Comparing gene inventories by assigning homology among distantly related genomes is not trivial [19]. The first challenge is to discriminate between genetic gains or losses and genes whose sequences are sufficiently divergent to escape detection. The problem is exacerbated by lineage specific gene or genome duplications, by varying rates of molecular evolution and by the sometimes fragile association between sequence similarity and the preservation of gene functions [20]. The second challenge is to recognize that reference genome annotations and data banks are fluid, even those for premier model systems. Therefore, this study uses sequence similarity searches for *Daphnia *genes against a number of different genomic databases for five reference species while intentionally setting low statistical cut-off values. By comparing *Daphnia *sequences to genes from four insect species and using *Caenorhabditis *for an outgroup, we point to functional classes that are shared with the insects. The related microarray data of Eads et al. and Shaw et al. in companion papers demonstrate that most of the sequenced *Daphnia *genes are differentially transcribed in a manner consistent with their putative functions, thus reinforcing their provisional annotations based on sequence alignments to genes from model insects. Our study details the comparative and functional characterization of *Daphnia *transcripts using well studied insects and a phylogenetic approach.

## Results

### Production and quality assessment of cDNA libraries

Equivalent non-normalized cDNA libraries were constructed from a genetically clonal *Daphnia *isolate sampled from a natural pond along the Oregon coast. The clone was cultured under growing conditions favoring parthenogenetic reproduction. Consequently, the animals were predominantly juvenile females, adult females and brood-carrying females with a small proportion of males.

The strength of conclusions derived from the comparative analysis of expressed gene sequences rests, in large part, on the quality of cDNA libraries. Therefore, we performed quality control tests on 768 randomly chosen cDNA isolates. The cDNA size distribution was determined by agarose gel electrophoresis of PCR amplified inserts. The average molecular weight of inserts sampled from the libraries was 825 bp. To assess the cDNA diversity within the libraries, we sequenced single pass 5' reads from the cDNA inserts. Of the 768 sequence reads, 619 were informative. Only four plasmids were void of inserts and the failed reads (19%) were the result of capillary failures of the sequencer. Following an assembly of the ESTs, unique sequences comprised 68% of the total, reflecting the relative abundances of specific cDNA within the non-normalized libraries. This number diminished to 43% with over twice as much sequencing effort (see below).

To assess potential contamination of cDNA clones derived from prokaryotes and mitochondria, and to measure the distribution of full length ORFs, we aligned the translated sequences to proteins in Genbank. Of the unique sequences, 50% matched Genbank entries with e-values < 1 × 10^-10^. A separate query of the NCBI non-redundant protein database identified 204 sequences with e-value scores < 1 × 10^-27^. A total of 34 ESTs (6%) were identified as mitochondrial gene transcripts. No ESTs were identified as non-*Daphnia*. Thus, the cDNA libraries are high quality with a high level of diversity and low levels of contaminant sequences.

To investigate whether the libraries contained full-length or nearly full-length inserts, sequences from 170 clones with high similarity to known proteins (Blastx < 1 × 10^-27^) were investigated for the presence of a translational start site. Of these sequences, 109 ESTs (64%) contained unambiguous open reading frames with an annotated ATG translational start site at their 5' end, and 44 ESTs (26%) did not contain an ATG that aligned with the start sites of corresponding database sequences. Of the remaining sequences, 7 ESTs (4%) were likely full-length because gapped alignments of the amino acids suggested poor evolutionary conservation at the N-terminus of the proteins, and 10 ESTs (6%) were unresolved because alignments failed altogether. We therefore estimate that 64–68% of the cDNAs are full-length, or close to full-length. This result may be an overestimate since many conserved genes within our non-normalized libraries encode for ribosomal proteins (34% of 170) which seldom have long transcripts. Indeed, the maximum length of investigated cDNA for open reading frames was < 2 kb, whereas the maximum length of PCR amplified inserts was nearly 3.5 kb. However, when the number of cDNA with and without annotated start sites were compared and sorted by their molecular weights, no association was found between the proportion of full-length transcripts and the size of cDNA, neither by including ribosomal genes (t = 0.39, df = 86; p = 0.70) nor by excluding these genes in the comparison (t = 0.19; df = 60; p = 0.85). A separate investigation of the consistency in our production of full-length or near full-length cDNA was conducted by calculating the proportions of sequences that shared nucleotides within the first 50 bases of the longest EST within contigs (see below for assembly of contigs). Of 233 ESTs forming 81 separate contigs, 202 (87%) shared the first 50 bp of the longest EST from each contig. These data suggest that the majority of the cDNA clones are near full-length.

### Analysis of EST sequences

In total, we produced 5' sequence reads from 1,648 cDNA isolates. In addition to the 768 randomly selected clones, 880 were selected on the basis of their transcription profiles in microarray experiments. After the removal of vector, poly-A tails and poor quality reads (Table 1), 1,546 high quality ESTs with an average size of 540 bp (SD = 188, min = 107, max = 852 bp) remained to be clustered (Genbank accession numbers EE681877-EE683416). The ESTs were assembled into 804 clusters (including 568 singletons) with an average of 1.93 sequences/cluster (SD = 3.95, min = 1, max = 95). After excluding clusters identified as mitochondrial DNA sequences, 787 nuclear genes remained. These non-redundant sequences are hereafter referred to as assembled sequences. We expect that some pairs of assembled sequences will be found to derive from the same locus, either due to excessive polymorphisms between alleles, or because of the alternative use of 5'-exons, or due to sequences from truncated cDNA clones that failed to overlap. However, given the high proportion of estimated full-length clones in the libraries, we anticipate the latter class to be small.

**Table 1:** 

Number of sequenced cDNA isolates	1,529
Number of sequences obtained:	1,648
Number of low quality sequences removed	82
Number of plasmids containing inserts <100 bp	20
Number of cDNA isolates with ESTs	1,435
Number of ESTs left to assemble	1,546
Number of assembled sequences (contigs + singlets)	804
Number of cDNA isolates represented by a single EST	612
Number of mtDNA gene clusters	17
or Number of mtDNA ESTs	132
Number of assembled sequences from nuclear genes	787

Sequencing and clustering statistics for cDNA isolates printed on microarrays. Based on these numbers, 43% of the elements are unique.

We investigated the proportion of assembled sequences that may be composed of alternative transcripts of the same genes by further clustering these sequences using more relaxed parameters (see methods). Forty-seven additional clusters were discovered. Eleven were composed of 2 assembled sequences that are likely allelic variants of the same genes, based on their matches to a single location in a preliminary draft assembly of the *Daphnia *genome sequence at the mid-point of the genome sequencing project (4 × coverage; deposited at wFleaBase). These allelic sequences were 84% to 97% similar to each other over a range from 77 to 665 overlapping nucleotides. By contrast, 21 of the additional clusters were of transcripts derived from duplicated genes or from conserved gene families, based on their matches to different locations in the draft genome sequence. In 14 cases, the clusters were composed of 2 sequences. Six clusters consisted of 3 sequences and a single cluster contained 4 similar sequences that shared 86–93% of their nucleotides in pair-wise comparisons. Overall similarities between sequences from closely related genes ranged from 62% to 93% among 220 to 807 overlapping bases. As expected for sequences originating from separate loci, their average similarity (85.5%) was significantly lower than that of allelic sequences (91%)(t = 2.02; df = 44; p = 0.01). Finally, 4 of the additional clusters were composed of paired splice variants from unique loci, while 8 clusters contained from 2 to 4 alternatively spliced transcripts from multiple loci. Therefore, the ESTs from this survey provided sequence tags for up to 787 new *Daphnia *genes, where some genes represent alternative transcripts or are closely related transcripts from duplicated genes.

### Functional annotation of assembled sequences

**Figure 1:**
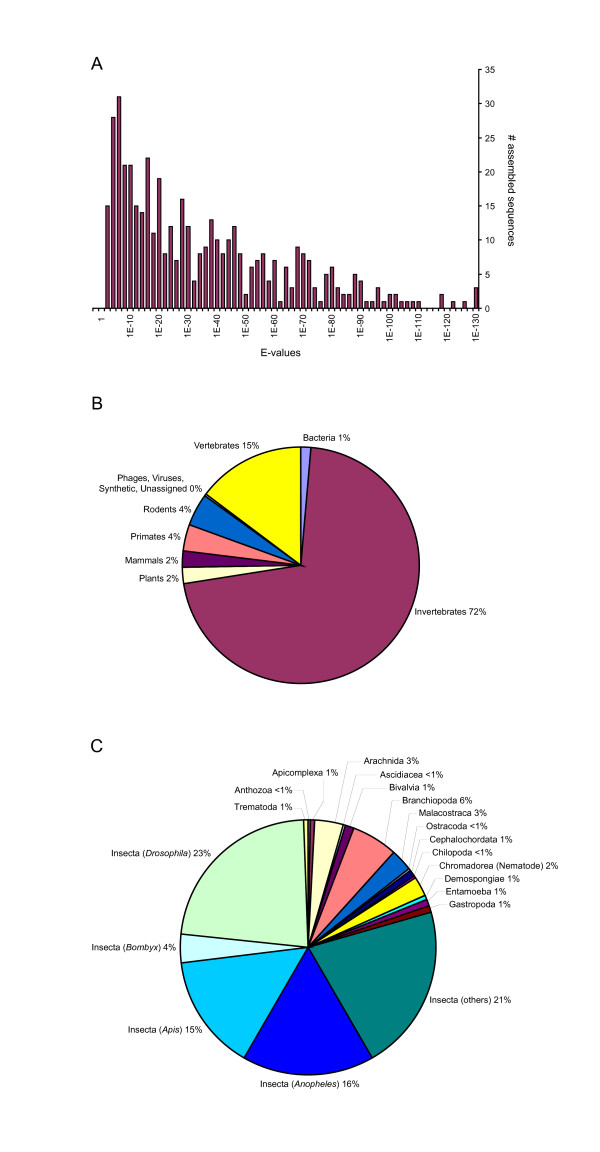
Results from Blastx searches of the assembled *Daphnia pulex *cDNA sequences against the NCBI non-redundant protein database. (A) Distribution of e-value scores. (B) Distribution of top matches against the NCBI taxonomic domains. (C) A more refined distribution of the best hits that were matched to protein sequences belonging to invertebrates.

Confident over the quality of the *D. pulex *cDNA libraries and EST sequences, we explored the range of likely biological or biochemical functions of the genes represented by the ESTs sequences by querying the NCBI non-redundant protein databank (NR) using Blastx [21]. Of the 787 assembled sequences, 452 (58%) matched at least one known protein with an e-value threshold of 1 × 10^-3 ^and a minimal value of 33 aligned amino acids (Additional file 1). The distribution of their e-value scores showed that 26% of matched sequences have scores < 1 × 10^-50^, while 79% have scores < 1 × 10^-10 ^(Figure 1a). Therefore, searches for putative homologues in the protein database gave strong to suggestive information regarding possible biological and biochemical functions. As expected, a survey of the distribution of best Blastx matches against the NCBI taxonomic domains showed that the majority (72%) of assembled *Daphnia *sequences matched best with those derived from other invertebrates (Figure 1b), whereas 25% of the highest scoring hits matched best with vertebrate sequences (including those from rodents, primates and other mammals). The cDNA libraries are free of contaminants, as only 6 assembled sequences (1%) matched bacterial proteins. A closer examination of the distribution of the best Blastx hits within the classes of invertebrates showed that 79% of 323 assembled sequences matched annotated proteins from insects (Figure 1c): 23% from *Drosophila*, 16% from *Anopheles*, 15% from *Apis*, 4% from *Bombyx *and 21% from other insects. This large insect constituency within the best Blastx matches is clearly a consequence of the limited representation of sequences from Crustacea in the databanks. A survey of the NCBI protein database revealed that out of 6,897,314 archived sequences, only 10,485 (or 0.1%) were from Crustacea. Only 19 assembled sequences (6%) best matched proteins from Branchiopoda, the class that includes *Daphnia*, while an additional 10 assembled sequences best matched proteins from other classes of Crustacea (Malacostraca, Ostracoda).

**Figure 2:**
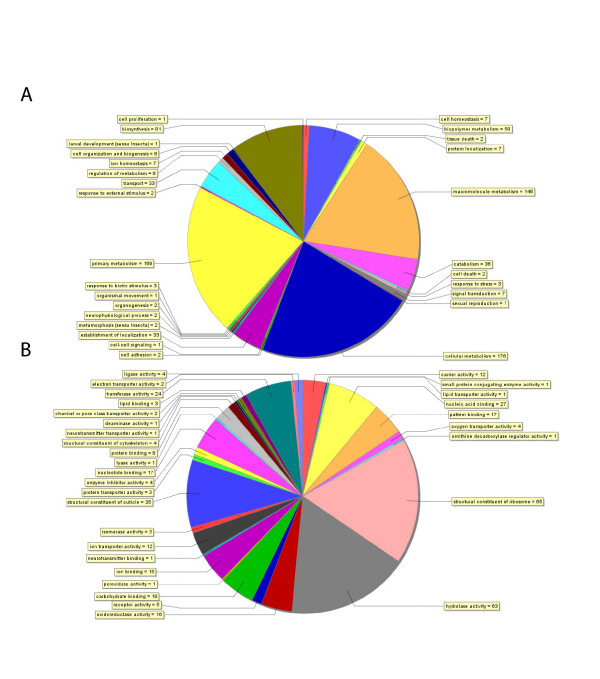
The distribution of gene annotations for the list of 787 *Daphnia pulex *ESTs based on results from Blastx searches against the NCBI non-redundant protein database. (A) The assignment of 799 annotations of biological process to 227 EST clusters from level 4 of the Gene Ontology. (B) The assignment of 371 annotations of molecular function to 288 EST clusters from level 3 of the Gene Ontology. Blastx queries recorded the best 5 matches with an E-value threshold of 1 × 10^-3 ^and a minimal value of 33 aligned amino acids. Gene Ontology (GO) terms were assigned to ESTs using Blast2GO [22] with the following configurations: Pre-eValue-hit filter 1 × 10^-3^; Pre-similarity-hit filter 2; Annotation cut-off 35; GO weight 5.

The assembled *Daphnia *sequences that matched annotated proteins from genetic model species were assigned Gene Ontology (GO) terms using Blast2GO [22]. Their putative functions spanned a spectrum of biological and biochemical processes (Figure 2a). A total of 227 assembled sequences were assigned 799 biological process terms from the fourth level of the GO. The predominant terms were for metabolic processes ascribed to 190 assembled sequences. These terms included cellular metabolism (22%), primary metabolism (21%), macromolecule metabolism (18%), biosynthesis (10%), biopolymer metabolism (7%), catabolism (5%) and regulation of metabolism (< 1%). From among these processes, 72 assembled sequences were annotated to involve protein biosynthesis, while 30 sequences involved the catabolism of proteins. Sixteen sequences were attributed roles in chitin metabolism, including chitinases and peritrophins. The next most predominant biological process terms were related to the localization of cellular components (establishment of localization, transport, protein localization), which were ascribed to 33 assembled sequences. Four of these sequences coded for genes involved in oxygen transport (hemoglobin). Thirteen of these sequences encoded genes with putative homologues in insects that specifically transport charged atoms like metals, of which seven were also ascribed the functions of cell and ion homeostasis (Figure 2a). Finally, the remaining assembled sequences with GO biological process terms were likely involved in cell communication (such as signal transduction, cell signaling and adhesion), development, and physiological processes that specify a response to external stimuli, stress and cell death.

A total of 288 assembled sequences were additionally assigned 371 GO molecular function terms (Figure 2b). One hundred and thirteen sequences were suggested to have catalytic activities that included hydrolase (17%), transferase (6%), oxidorectuctase (4%), ligase (1%) and isomerase activities, among others. Another 105 assembled sequences were likely involved in structural activities. Their GO terms included structural constituent of the ribosome (18%), structural constituent of the cuticle (9%) and structural constituent of the cytoskeleton (1%). Indeed, *Daphnia *genes matched to 67 of the total number of 194 listed *Drosophila *ribosomal components. The next major functional class was represented by 86 sequences putatively involved in binding, which included 27 sequences coding for nucleic acid binding proteins, 18 carbohydrate (also listed as pattern and chitin) binding proteins, 17 nucleotide binding proteins, and 15 proteins that bind ions (calcium, zinc, iron). The remaining 11 sequences within this class were protein or lipid binding. The final major functional class contained 26 assembled sequences assigned to have transporter or carrier activities; twelve sequences were annotated as ion transporters.

A number of assembled sequences likely encode conserved proteins involved in gene regulatory functions. *Daphnia *genes with such potential functions based on sequence homologies included 19 sequences involved in transcription regulation and 14 sequences with translational regulator activity (Additional file 2). Examples of regulatory genes involved in arthropod development included a putative homologue to *maf-S*, which is a basic-leucine zipper (bZIP) transcription factor in *Drosophila *that is required for the development of pharyngeal structures [23]. A *Daphnia *sequence also matched closely to the *Dorsal switch protein 1 *(*Dsp1*) gene that regulates a number of homeotic genes in *Drosophila *[24] and is therefore involved in many developmental pathways. The putative homologue to the fly gene *shaggy *(*sgg*) was identified, which is part of the Notch, Wnt and Smoothened signaling pathways. Interestingly, two other regulators of the Notch signaling pathway called *Cdc42 *[25] and *neurotic *(*nti *or *O-fut1*) were also identified (Additional file 3). The fly gene *nti *is specifically required for the proper localization of *Notch *at the cell surface [26], is essential for the physical interaction of *Notch *with its ligand *Delta*, and is an essential component for neurogenesis [27]. A second gene involved in neurogenesis was identified as homologous to *similar to Deadpan *(*Side*), which is a basic helix-loop-helix (bHLH) transcription factor. A *Daphnia *gene was also matched with the fly zinc-finger-C4 transcription factor (ZnFC4) called *ftz transcription factor 1 *(*ftz-f1*), which coordinates stage-specific responses to the steroid hormone ecdysone during metamorphosis [28] and directs key developmental events at the transition between prepupal and pupal stages of *Drosophila *development [29]. The putative functions for the other identified transcription regulators (Additional file 2) included the regulation of mitotic progression (the TFIIH transcription factor *Cdk7*) and important roles during gametogenesis (*Rab11, bic*, and the C2H2-zinc finger transcription factor *Meics*). Other putative transcription factors included genes matching *CG1876I *that contains a helix-turn-helix (HTH) DNA-binding motif, *CG18619 *that contains a bZIP DNA-binding motif and *CG3224 *that contains a putative zinc-finger DNA-binding motif. Finally, among the regulators of translation (Additional file 2), *Daphnia *genes matched to 7 of the total number of 24 listed *Drosophila *translational elongation genes, yet matched only to 2 of the 58 listed translational initiation genes in flies. Their putative functions include DNA repair (*RpLP0*), autophagic cell death (*eIF-5A*, *Ef1gamma*), immune response (*RpS6*, *Thor*), regulation of cell growth (*Thor*) and germ-line stem cell division (*piwi*).

To discover *Daphnia *genes that may jointly participate in conserved biological processes or within gene interaction networks, we investigated GO classes that were highly represented within our list of putative homologues to fly proteins. Significant functional groupings of *Daphnia *genes were expected, because more than half of the sequenced ESTs were chosen based on their differential expression patterns in separate microarray experiments examining developmental differences among males and females, juveniles and adults, and toxicological responses to metals (Eads et al. submitted; Shaw et al. submitted). Seventeen genes were identified as candidates for gametogenesis (Additional file 4); the majority of these loci are involved in oocyte development in flies. *Daphnia *loci matching the fly genes *mago*, *Rab11*, *chic*, *Tm1*, *tsu *and *bic *may play conserved evolutionary roles in specifying the anterior-posterior axis of the oocyte. All 6 genes save *bicaudal *(*bic*) coordinate to assemble the pole plasm at the posterior end of the *Drosophila *oocyte by localizing maternally derived transcripts for *oskar*. Moreover, *mago*, *Tm1 *and *Rab11 *are known to interact in genetic screens [30], while a two-hybrid-based fly protein interaction study [31] implicates *tsu *and possibly a translational elongation *RpLP1 *homologue (Additional file 3) within this network. One other *Daphnia *gene with weak sequence similarity to *Sop2 *may have transport functions during oogenesis [32] and two genes similar to the signaling gene *Cdc42 *and to *tsr *were respectively identified, which are involved in follicle cell development [33,34]. Other genes similar to *snf*, *RpS3A *and *sgg *in flies are also candidate for oogenesis. Only two genes from our survey have known homologues in flies that function in spermatogenesis. The gene *Act5C *has a role in sperm individualization [35] and *Meics *is associated with central spindle and mid-body microtubules during meiosis [36]. The *chic *gene in flies is important in gametogenesis for both sexes [37]. These *Daphnia *genes sharing sequence similarities with *Drosophila *loci, which are known to coordinate conserved developmental processes, are prime candidates for functional investigations of early crustacean development.

In contrast to genes that participate in biological processes that are shared between Crustacea and Insecta, gene families that have expanded in *Daphnia *compared to insects may be indicative of new gene functions linked to their specific biology and ecological setting. Therefore, we identified GO classes that were overrepresented within our dataset compared to the Gene Ontologies for the *D. melanogaster *proteome using Fisher's exact test, and counted multiple assembled sequences that matched to unique *Drosophila *proteins (Additional file 1). Some lineage expansions seemed to occur primarily by tandem duplication, while other radiations implied interesting functional specializations or innovations. Among the 53 *Daphnia *sequences that were provisionally annotated as cuticle proteins and genes involved in chitin metabolism and molting, only 13 singularly matched to a fly gene (Additional file 5). In most cases, 2–4 assembled sequences matched to the same protein in the fly genome. Yet in another case, 15 sequences matched with the *D. melanogaster *gene CG6305, which contains an insect cuticle protein domain. Because we could not produce a reliable sequence alignment for all 15 assembled sequences, we conclude that the observed gene expansion was not an artificial result from inadequate clustering of redundant ESTs. Yet, from the pairwise comparisons of these 15 sequences, alternative splice variants for three genes were identified: Contigs 20 and 180 showed >90% sequence similarity, Contigs 23, 241 and 257 were >94% similar, and Contigs 19 and 24 were >85% identical over shared exons. Additional cDNA sequence data aligned to a completed genome sequence assembly for *Daphnia *is needed to confirm that cuticle proteins are expanded gene families compared to insects. However, our study also uncovered clearer examples of gene expansions.

Unlike insects, which have three ferritin genes that play important roles in iron homeostasis of cells (Fer1HCH, Fer2LCH) and of organelles (Fer3HCH), the annotation of *Daphnia *sequences revealed seven assembled sequences with strong matches to *Drosophila *ferritin proteins (Table 2). Singlet 73 showed a strong match to the *Drosophila *Fer1HCH protein via a Blastx alignment (bit score = 137), but was poorly matched to crustacean sequences, even to a *D. pulex *ferritin sequence within Genbank (AJ245734; bit score = 71). The remaining six *Daphnia *sequences, plus the Genbank entry, all aligned best to other crustacean sequences and to the single Fer3HCH locus of *Drosophila*. Therefore, Singlet 73 represents the first sequence of an orthologous crustacean Fer1HCH gene. This result was verified by constructing a phylogeny using representative insect and crustacean protein sequences and by including additional *Daphnia *ferritin-like sequences that were extracted from an ongoing *D. pulex *cDNA sequencing project (Colbourne et al. in preparation).

**Table 2:** 

*Daphnia *ID	*Drosophila *gene ID	*Drosophila *gene name	FlyBase ID	% similarity	E-value	Bit score
Singlet 73	CG2216	*Fer1HCH*	FBgn0015222	39.81	4.00E-33	137
Contig 91	CG4349	*Fer3HCH*	FBgn0030449	39.66	6.00E-27	117
Contig 26	CG4349	*Fer3HCH*	FBgn0030449	38.22	8.00E-23	103
Contig 138	CG4349	*Fer3HCH*	FBgn0030449	40.61	1.00E-22	103
Contig 217	CG4349	*Fer3HCH*	FBgn0030449	40.16	4.00E-14	74.7
Contig 40	CG4349	*Fer3HCH*	FBgn0030449	33.75	2.00E-08	54.7
Contig 42	CG4349	*Fer3HCH*	FBgn0030449	26.25	2.00E-06	47.8

*Daphnia *genes annotated as candidates for iron ion homeostasis based on sequence conservation with *Drosophila *genes with known functions. Contig 26 and 138 are two alleles from the same locus. Contigs 40, 42 and 91 are also sequence variants from the same locus.

**Figure 3:**
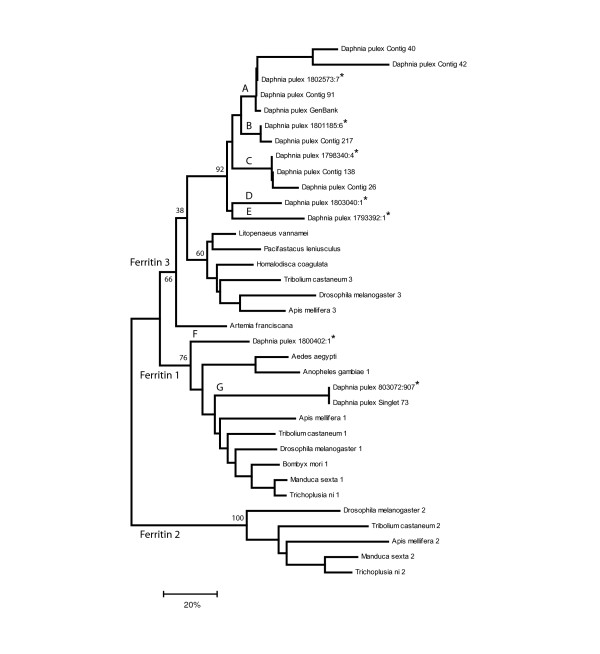
Lineage specific expansion of the *Daphnia pulex *ferritin genes. Neighbor-Joining (NJ) tree inferred from the deduced amino acid sequences of the *Daphnia *ferritin genes, including loci from *Drosophila melanogaster *plus other representative insect and crustacean amino acid sequences obtained from the NCBI and FlyBase protein sequence repositories. Ferritin 1 group contains insect and *Daphnia *Fer1HCH gene(s). Ferritin 2 group only contains insect Fer2LCH loci and the Ferritin 3 group contains insect and crustacean Fer3HCH genes. The amino acid sequence alignment was obtained by using t-coffee [72] and is available by request. The NJ tree was constructed using MEGA3 [73] using the Poisson correction for calculating the distance matrix. The bootstrap support values are shown at the main branch nodes of the tree, which are derived from 1000 pseudo-replication of the data. *D. pulex *sequences denoted by * were obtained from an ongoing cDNA sequencing project by the Joint Genome Institute and the *Daphnia *Genomics Consortium (Colbourne et al. in prep) and are deposited in Genbank under accession numbers DQ983425-DQ983438. GenInfo (GI) accessions for all other sequences: 6946692; 61744051; 26006755; 46561742; 91081285; 87083910; 66504201; 1807496; 13195275; 55242312; 66524157; 91077442; 24651358; 95702694; 18031707; 62722854; 6409191; 91077446; 66524161; 7272336; 62722856.

The Neighbor-Joining tree of 35 aligned amino acid sequences clustered the pancrustacean ferritins into three main groups (Figure 3). Ferritin 1 contained insect genes plus two cDNA from *Daphnia *libraries; the Singlet 73 amino acid sequence was identical to a sequence extracted from other cDNA libraries (branch G, Figure 3), while branch F was a unique gene that stemmed at the base of the group. The ferritin 2 group was solely composed of insect genes. However, both crustacean and insect sequences clustered into the third group containing the insect ferritin 3 genes. Although this group contained single copies of the insect genes, the *Daphnia *genes were further subdivided among five branches representing distinct ferritin 3 loci within the *D. pulex *genome. Branches D and E were gene sequences derived from the other libraries and showed 45% sequence divergence from each other, while branches A, B, C each contained at least one sequence from this present study. Further investigations indicated that the multiple sequences clustering within branches A, B and C are different alleles of the same locus. Given that insects and more distant outgroups have only three ferritin genes, *Daphnia *ferritins clearly expanded to include *possibly *one additional ferritin 1 locus and *minimally *four additional ferritin 3 genes.

### Gene conservation between the crustacean *Daphnia *and the true insects

**Figure 4:**
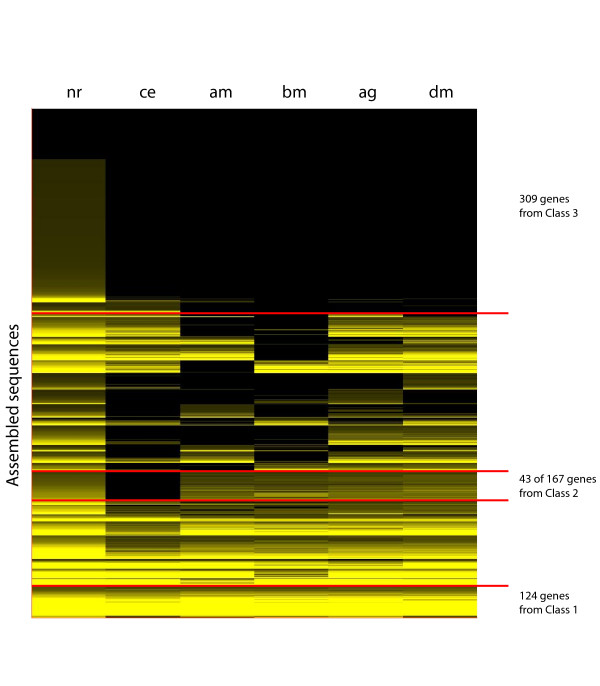
The clustering of the *Daphnia pulex *assembled ESTs based on their matches to genes from multiple databases, obtained from tBlastx searches against NCBI UniGene sets for *Caenorhabditis elegans *(CE)(build #23), *Bombyx mori *(BM)(build #7), *Apis mellifera *(AM)(build #5), *Anopheles gambiae *(AG)(build #29), *Drosophila melanogaster *(DM)(build #37) and from Blastx searches against the NCBI non-redundant (NR) protein database. The color intensity is proportional to the Bit Score, which ranges from <50 (black) to 535 (bright yellow). Three classes of interesting genes are indicated (see text).

To examine the conservation of genes represented by the EST sequences across Pancrustacea, we used tBlastx to match the *Daphnia *assembled sequences to the NCBI UniGene sets for *Drosophila melanogaster*, *Anopheles gambiae*, *Bombyx mori*, *Apis mellifera *and included *Caenorhabditis elegans *for an outgroup. The assembled sequences were clustered into 27 groups, based on the strength of these sequence matches across these taxonomic data sets. This arrangement identified a variety of gene classes that share patterns of sequence conservation (Figure 4). The first class of interest was composed of 124 genes (16%) that are conserved equally among all species included in this study. This class was mostly enriched by genes that participate in protein metabolism including protein modifications (67 genes to GO:0044267; p = 4.8 × 10^-7^) plus 32 genes that were likely involved in cellular metabolism (total of 99 genes to GO:0044237; p = 3.2 × 10^-8^). Other genes enriched within this class included transcriptional regulators (12 genes to GO:0045449; p = 2.4 × 10^-3^). The second class of interest was composed of *Daphnia *sequences that matched a nematode protein plus at least one insect locus (183 genes) and others that had no matches to nematode proteins yet matched to at least one insect proteome (167 genes). Therefore, 21% of the sequences were derived within the Pancrustacea – thus shared by *Daphnia *plus at least one insect in our set – or lost within the nematode. Especially noticeable were 43 assembled sequences that had no detectable homologues in worms and were uniformly conserved across the four insects (Figure 4). Genes that were absent in nematodes were enriched with structural constituents of the cuticle (26 genes to GO:0042302; p = 1.1 × 10^-12^) and loci having serine-type endopeptidase activity (10 genes to GO:0004252; p = 3.6 × 10^-4^), of which 8 genes were annotated as also having chymotrypsin activity (GO:0004263; p = 0.003). The third class of interest consisted of 309 genes (39%) that had no matches to insect proteomes. At face value, this result suggests that these orphaned genes are unique to *Daphnia *or Crustacea; either they have been lost in the insects – as suggested by 17 *Daphnia *genes (2%) showing sequence similarity to proteins in the nematode database – or acquired by *Daphnia *or Crustacea since diverging from their last common ancestor.

**Figure 5:**
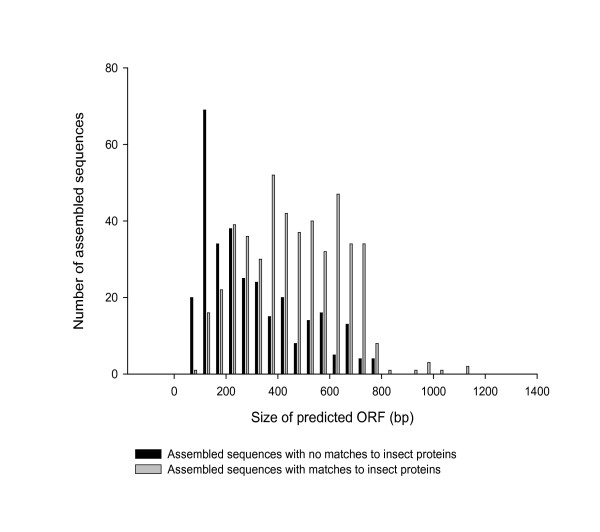
The distribution of predicted open reading frames (ORFs) for two classes of assembled EST sequences for *Daphnia pulex*. Black bars represent genes with no detectable matches to insect proteomes. Grey bars represent genes with matches to insect proteins based on Blastx searches.

There are many potential sources of errors that can inflate our estimate of the fraction of unique *Daphnia *genes compared to the selected pancrustaceans. For instance, sequences may fail to align for technical reasons. This may occur if the *Daphnia *sequences included untranslated regions (UTR) of the cDNA and not the coding regions. Indeed, the mean size of predicted open reading frames (ORFs) within this class differed significantly from that of genes having sequence matches to insect proteomes (t = 12; p < 0.0001; df = 785). For example, over half of the assembled sequences with no matches had ORFs smaller than 225 bases compared to 16% of matched sequences (Figure 5). Therefore, the trivial explanation that these sequences were mostly UTR cannot be dismissed for a large fraction of these genes. Other technical explanations for the absence of matches include genes that had not been annotated or included in the insect UniGene sets. Further analysis by aligning the non-matching sequences to all predicted *Drosophila *gene translations uncovered 9 additional matches with e-values ranging from 4 × 10^-3 ^to 2 × 10^-10^. Another 4 *Daphnia *sequences were found to have matches to *Drosophila *proteins, based on tBlastx searches against the full genome sequence (e-values ranging from 4 × 10^-3 ^to 9 × 10^-29^). Finally, given the tremendous evolutionary divergence between *Daphnia *and insects, matches may not have been detected from loci that are not under similar evolutionary constraints. We are unable to investigate this last point with the current data.

It was previously shown that gene preservation is correlated with gene function. In particular, correlations have been found between the level of gene conservation and sex-biased gene expression among insects [38,39]. There is reason to believe that such correlations are extended to other biological functions. In comparing 787 *Daphnia *assembled sequences to those of insects, 39% of the genes were characterized as orphans because no sequence matches were detected. Interestingly, the orphan genes were not randomly distributed among the gene expression classes. Three specific observations were made by incorporating the gene expression datasets (Table 3). First, 47% of male-biased genes did not match insect proteins compared to only 22% of female-biased genes. The two fold difference in sequence similarity among the sex-biased genes in *Daphnia *is consistent with differences seen among the insects, reflecting the overall accelerated evolution of male reproductive genes [40]. Second, 46% of metal responsive genes did not match insect proteins compared to only 34% of non metal responsive genes. Third, genes that were responsive to metals and not sex-biased included the greatest proportion of orphans (50%), whereas genes that were female biased and not responsive to metals included the fewest (12%). These results suggest that lineage specific genes are correlated with certain biological functions associated with an organism's ecological challenges.

**Table 3:** 

Response to metals	Up in males	Up in females	No change between sexes	Total
Responsive to cadmium and/or arsenic	45% (85/188)	42% (25/59)	49% (47/96)	46% (157/343)
No change in both metals	48% (95/197)	12% (13/112)	33% (44/135)	34% (152/444)
Total	47% (180/385)	22% (38/171)	39% (91/231)	39% (309/787)

The percentage of *Daphnia pulex *assembled EST sequences with no matches to insect proteins, partitioned by their differential expression patterns in experiments designed to detect transcriptional differences between the sexes (Eads et al. submitted) and genes responding to toxic metal exposure to cadmium and arsenic (Shaw et al. submitted, and in prep). A 5% false discovery rate is applied to all three experimental results. The number of orphan genes over the total number of genes within the partition is indicated in parentheses.

## Discussion

Diversity in the gene complement of species arises from the expansion of shared ancestral gene families, the loss of existing genes, or the acquisition of newly invented genes [12,41-43] and can account for lineage specific innovations. It is estimated that nearly half of the paralogous gene families within eukaryotic genomes originated by lineage specific gene expansions; many are related to an organism's unique mode of life [12]. For example, the evolution of disease resistance and of self-incompatibility in plant mating systems can partly be attributed to the radiation of novel receptor-like kinases within the plant genome [44]. In *Drosophila*, the trypsin-like serine proteases have expanded to 178 genes [45], suggesting important novel defenses by the fly immune system. Odorant receptors form the largest recorded nematode-specific gene family expansion – numbering ~800 genes compared to 60 genes in flies [46] – which suggests the importance of chemosensing in the soil environment. A similar genomic inventory for a branchiopod crustacean genome will soon be made available by the *Daphnia *Genomics Consortium to ultimately contrast the evolutionary diversification of arthropod genes in relation to the aquatic and terrestrial habits of these animals. Yet, crustaceans and insects also share many key biological features due to their common ancestry as members of the Pancrustacea. This study presents the results of a first investigation into the sequence conservation and putative function of *D. pulex *genes, which are identified by sequencing a set of 1,648 cDNA isolates that were interrogated in three microarray studies. From clustering the ESTs, we characterize 787 *Daphnia *loci based on their sequence similarity to genes within a variety of databases, including those for the insects *Bombyx*, *Apis*, *Anopheles, Drosophila*, and for the nematode *Caenorhabditis*.

### Shared genes

In this study, we characterize two non-normalized cDNA libraries from a clonal population reared under standard laboratory conditions. As a result, the diversity of biological processes and molecular functions represented among the sequenced *Daphnia *genes is relatively modest. Almost one quarter of the genes are likely involved in metabolic processes. More than one quarter of the genes are predicted to have catalytic or structural activities. Although a large fraction of arthropod genomes is composed of genes having these basic cellular functions (35–40% of *Drosophila *genes for instance), the diversity of transcribed genes discovered in *Daphnia *would be augmented by creating libraries from animals under a variety of environmental conditions. Yet, our libraries do contain cDNA from daphniids of mixed life stages, including gravid females, embryos, juveniles and a small number of males. Therefore, some *Daphnia *genes have sequence similarity to insect proteins associated with reproduction, development and growth. These include regulatory genes like *Dsp1*, which operates in patterning the developing fly embryo by acting as a corepressor of the transcriptional regulator Dorsal protein [47]. Under different circumstances, *Dsp1 *can also act as an activator or repressor of thorax-group and polycomb-group homeotic genes in *Drosophila *[24]. Because all known homeotic targets of *Dsp1 *are conserved in sequence and function in metazoans, *Daphnia*'s putative orthologue is likely to share this regulatory function. As expected, this gene transcript is enriched in pregnant females compared to males in *Daphnia *microarray experiments (Additional file 2). Putative homologues to three regulatory genes (*sgg*, *Cdc42*, *O-fut1*) within the Notch signaling pathway are identified, which is one of a small number of signal transduction pathways that are highly conserved in insects and throughout animal evolution. The gene *sgg *is a point of convergence between the Notch and Wnt/wingless signaling pathways [48]. We predict that further sequencing of *Daphnia *cDNA will uncover more genes operating within these and other conserved signaling mechanisms: nuclear receptors, Sonic Hedgehog, receptor tyrosine kinases, JAK/STAT, and BMP/TGF-beta. An example is a homologue to the *ftz-f1 *nuclear hormone receptor that is also found on the microarray. Like all arthropods, *Daphnia *growth is synchronized with molting and the regeneration of the cuticle, which is governed by pulses of ecdysteroid hormones. Although one isoform of *ftz-f1 *is a transcriptional regulator of the embryonic segmentation gene *fushi tarazu*, a second isoform is necessary for larval molting in *Drosophila *and its premature expression results in the disruption of the epicuticle, suggesting that targets for this transcription factor in flies include genes involved in cuticle formation [49]. The function of this gene is conserved in *Caenorhabditis*, where it is also required for epidermal development and regulates molting [50]. No differential expression is observed on the microarray for the signaling genes discussed above (Additional file 3). Overall, our survey of *Daphnia *ESTs uncovers genes expected to be present in crustacean genomes based on their important regulatory roles in conserved cellular and developmental processes.

Roughly half of the cDNA isolates that are sequenced for this study are chosen based on their differential expression patterns between males and females and on their responses to toxic metals. These experimental conditions reflect two research interests of our labs involving *Daphnia*: the genetic basis of environmental sex determination and cyclical parthogenesis, and understanding how populations adapt to environmental change in aquatic habitats including industrial pollutants. Therefore, we expect that the provisional annotations of 787 assembled sequences include a fraction of genes sharing functional attributes that would in part be shared with other arthropods and others that are more reflective of *Daphnia*'s unique biology. Certainly, 17 genes are candidates for gametogenesis. The majority of these genes play significant roles in oogenesis. In particular, six sequences have strong matches to conserved genes that specify the oocyte polarity and four loci are known to genetically interact in flies. By contrast, only three of these 17 *Daphnia *sequences match genes that are known to function during spermatogenesis in *Drosophila*. This discrepancy between the numbers of sex specific transcripts for genes involved in reproduction is likely caused by the small representation of males within the *Daphnia *cultures used to create the cDNA libraries. Equally impressive are the large number of cuticle proteins identified.

### Expanded protein families

In the comparative study of the *Anopheles *and *Drosophila *proteomes [51], cuticular proteins were noted to be particularly active in their lineage specific expansions and deletions. This present study identifies 53 sequences that are either structural components of the cuticle or involved in chitin metabolism. Their abundance within our dataset is a consequence of their transcriptional responses to the microarray experiments; all but four of the assembled sequences were differentially regulated on the arrays. Yet as noted in the comparisons between the two dipteran insects, many *Daphnia *sequences share similarities to single loci within insect genomes. Among these cuticle loci are 15 assembled sequences that are best aligned to a single *Drosophila *gene when compared to the rest of the fly proteome. Alternative transcripts account for only 4 sequences. Thus, no less than 11 loci remain as possible representatives of a large lineage specific gene expansion of *Daphnia *cuticle genes. Further investigations are obviously required to verify this finding, including more thorough sampling of the *Daphnia *transcriptome and functional data such as *in situ *hybridizations to support the notion that these genes may have contributed to biological innovations. Regarding the 15 transcripts on the current array, all but one is enriched in males compared to females, and with the addition of differential expression patterns under metal stress, these transcripts can be grouped into five separate expression profiles.

A first compelling case for lineage specific gene expansion is made by investigating the diversity of ferritin genes within the *Daphnia *ESTs. Except for *Aedes aegypti*, their number of ferritin genes are evolutionarily conserved [52]. Ferritins are the principle iron storage proteins for nearly all animals, and their abundance within cells is controlled in part by iron-regulatory proteins that interact with iron-regulatory elements (IREs) within alternatively spliced 5' UTRs of certain mRNAs [53,54]. In insects, ferritins consist of a heavy-chain homolog (HCH) and a light-chain homolog (LCH) forming heterodimers that function in the secretory pathways of cells, and which also appear to act as iron transporters [52]. The genes encoding the subunits (Fer1HCH, Fer2LCH) are positioned in the *Drosophila *genome in a back-to-back orientation, enabling coordinated regulation of their transcription [55]. This feature is conserved in all insects studied thus far [52]. Except in *Bombyx*, which has IREs within the UTRs of both subunits, insect IREs are predominantly localized to the Fer1HCH locus. Recently, a third *Drosophila *ferritin (Fer3HCH) has been described that controls iron homeostasis of the mitochondria, yet its transcription is not responsive to iron treatment [56]. As in humans and mice, the gene is predominantly expressed in adult testis. Our phylogeny of *Daphnia *ferritin gene transcripts uncovers six or seven distinct *Daphnia *loci (Figure 3). The branch F locus cannot be unequivocally included as part of the ferritin expansion until a description of the gene is available based on its alignment to the genome sequence. However, all of the other loci are defined based on their sequence alignments to distinct genome scaffolds assembled at this point in the *Daphnia *genome sequencing project (not shown).

Branch G of the ferritin phylogeny represents the first characterized crustacean orthologue to the insect Fer1HCH genes. Following naming conventions, we designate this gene as Dpu_Fer1HCH. The gene has four introns and is the only *Daphnia *ferritin on the array showing differential expression for all three experimental conditions; its transcripts are enriched in males, depleted when exposed to cadmium and enriched when challenged by arsenic (Eads et al. submitted; Shaw et al submitted and in prep). Therefore like the insect subunit, this locus responds to metal ion treatments. The other five *Daphnia *ferritin genes are homologous to the insect Fer3HCH loci and are arranged within a monophyletic cluster, suggesting that they originate from a series of gene duplications. Indeed, each has retained two introns, despite showing amino acid sequence divergences from 14 to 56%. These genes are designated Dpu_Fer3HCH-1 to Dpu_Fer3HCH-5. Regrettably, expression data is not available for genes representing branches D and E. However, the microarray results show that the remaining three loci differ in their transcriptional responses to the experimental treatments. Like the *Drosophila *Fer3HCH gene, elements on the array whose sequences cluster within branch C do not respond to metals, yet unlike the fly gene, Contig 26 transcripts are enriched in females. By contrast, a single element on the array representing branch B is enriched in males, and all 4 cDNA elements from this gene respond to arsenic treatment. These differences are likely the result of undetected splice variants. Lastly, all elements representing branch A show elevated expression patterns when treated with arsenic, yet no sex specific expression is detected. These additional observations strongly support the existence of a Fer3HCH gene expansion that diversified within a crustacean lineage leading to *D. pulex*. A homologue to the insect Fer2LCH genes has yet to be discovered in Crustacea.

### Orphan genes

The present study is a preliminary annotation of the emerging *D. pulex *genome using comparative and functional data to predict the fraction of genes unique to *Daphnia*. Aside from gene expansions that are suggestive of adaptations specific to aquatic environments, accounts of orphan genes (defined here has having no matches to insects) can offer equally important insights into crustacean biology from the perspective of a class of genes that usually has the shortest average lengths and are most rapidly evolving [51,57]. Our searches using Blastx of 787 assembled sequences against the proteome of four insects suggest that ~68% of the *Daphnia *genes are shared with at least one insect. Less than half of these show matches to the individual protein databases of *Bombyx*, *Apis*, *Anopheles *and *Drosophila*. This discrepancy is likely a combined effect of incomplete datasets and of lineage specific gene losses among the insects. Taking into account associated matches to proteins from our chosen outgroup (*Caenorhabditis*), we discover that 21% of the *Daphnia *genes are either derived within Pancrustacea or lost within the nematodes. Those genes that are uniformly conserved across all four insect species are primarily cuticle proteins and serine proteases having trypsin activity. These gene families are also listed as two of the top 20 most significant expansions or reductions between the *Anopheles *and *Drosophila *proteomes [51], which diverged some 250 million years ago. It is tempting to speculate that, in both crustaceans and insects, a fraction of gene families are equally active in their evolutionary diversification. Such gene families would be candidates for detailed investigations leading to a better understanding of how Pancrustacea succeeded in exploiting its range of ecological settings.

A careful evaluation of assembled sequences showing no matches to the insect proteomes suggests that ~1/3 of the genes are either derived in crustaceans or lost within insects. This estimation is admittedly from a very limited sampling of the total number of *Daphnia *genes and is derived from sequencing non-normalized cDNA libraries that were created under standard laboratory conditions and interrogated by microarrays. Although this fraction cannot be extrapolated to the genome, it is comparable to findings from other taxa. In *D. melanogaster*, 10% of the genes have homologous best hits in non-insect species plus 19% have no homologous hits to other species, while the combined estimate for *A. gambiae *is 21% [51]. Within the nematodes, which diverged ca. 600 mya, 23% of the genes are estimated to be unique to species [14]. Of course, the fraction of species specific genes declines dramatically when evaluating close allies; comparing two *Caenorhabditis *species reveals that 4% of their genes are unique [58], and the mouse gene set differs from the human set by only 1% [59]. A future investigation of a larger *D. pulex *gene collection against an equivalent dataset for the congener *D. magna *[60] will help define the true estimate of species specific genes in *Daphnia*. Additional contributions of EST data for non-branchiopod crustaceans will further define the crustacean proteome and shed light on the biological factors that led to the group's divergence from insects. However, the sequences presented by our present study are accompanied by expression data from three microarray experiments, which authenticate the orphan sequences as genes and support the notion that ecological factors are more likely to contribute to sequence and functional divergences among genomes.

Combining the gene expression data obtained by Eads et al. (submitted) and Shaw et al. (submitted, and in prep) for the 787 assembled sequences reveals that the majority of genes with sex biased expression, including developmental and regulatory loci, do not respond to the cadmium and arsenic metal toxicity. A clear example is provided by genes predicted to regulate translation. All 15 genes save two are differentially expressed in males versus females and only three genes also show transcriptional responses to metal toxicity (Additional file 2). We find that this class of sex biased genes proportionally contains the fewest orphans. The relatively larger number of sequences with matches to the insect proteome suggests that genes functioning during development and reproduction are generally well conserved between crustaceans and insects. Further work is required to elucidate crustacean and *Daphnia *specific components of these central processes. By contrast, nearly half of the *Daphnia *genes that respond to metals, but show no differences between the sexes, are likely absent in insects. This is explicable in light of the fact that metal exposure is an ecological stressor that varies between aquatic and terrestrial environments [61], which has catalyzed the evolution of certain protein types (cuticles, iron metabolism, defense) to increasingly specialized functions. The extent to which ecology has shaped the genome organization of pancrustaceans is an important future direction for research. For example, the mosquito *A. gambiae *spends part of its larval stage in water; by comparing genes differentially expressed during this stage to expression patterns in *D. pulex*, it may be possible to examine the effects of an aquatic lifestyle on the expression of particular protein families.

## Conclusion

This work investigates the sequence preservation and expansion of genes from the crustacean *D. pulex *compared to insect proteomes, based on the analysis of 1,546 ESTs that represent 787 unique transcripts. Our sampling of cDNA from this emerging genomic model species reveals sequences that have largely been conserved in both groups representing arthropods evolving in water or on land. Genes that function for reproduction, regulation of cellular processes and development are identified; some are known to genetically interact in the model insect species *Drosophila*. This provisional annotation of *Daphnia *sequences is further verified by companion studies using cDNA microarrays to examine transcription in males, females and embryos (Eads et al. submitted) and under toxic metal stress (Shaw et al. submitted, and in prep). Here we identify cases of lineage specific gene family expansions by a series of gene duplications. For instance, there are as many as seven distinct ferritin loci indicated by cDNA and genome data, including a crustacean orthologue to the insect Ferritin 1 locus and a monophyletic grouping of five Ferritin 3 genes. Finally, our results suggest that, as we study the genomes of organisms distantly related to the classic model laboratory organisms, the majority of unknown genes will be functionally linked to the organisms' ecology. Compared to the gene sets showing differential expression among developmental stages, we observe that sets responding to ecological stress contain a greater proportion of loci with no sequencing similarity to previously characterized arthropod genes. A comprehensive inventory of putative orthologs, orphan genes, and lineage specific gene expansions coupled with functional genomics data will provide important insights into genomic changes that led to the adaptive radiation of crustaceans.

## Methods

### cDNA library construction and quality assurance

For the purpose of creating a collection of cDNA for printing onto microarrays, a clonal isolate of a *D. pulex */*D. pulicaria *hybrid (called log52) was cultured under standard laboratory conditions by Jim Haney (University of New Hampshire) within a large, aerated, 200 liter container of filtered lake water by feeding a concentrated monoculture of green algae (*Scenedesmus acutus*). Animals at all life stages were harvested and immediately processed. Total RNA was isolated using Trizol reagent (Invitrogen Life Sciences) and was subsequently purified using the RNeasy protocol (Qiagen). The cDNA libraries were constructed by Darren Bauer and Kelley Thomas (University of New Hampshire) using the Creator SMART (Clontech) system by following the manufacture's instructions. The cDNA was ligated into the pDNR-LIB vector supplied by Clontech.

To control for bias towards smaller fragments inserting during the ligation of cDNA into plasmids, reaction were performed on four cDNA size fractions. Size fractionation was performed as per the SMART cDNA protocol using the CHROMA SPIN-400 column. The column was prepared for drip procedure by inverting several times to completely resuspend the gel matrix and storage buffer was drained by gravity flow. Seven hundred microliters of column buffer were added to the column and allowed to drain out, then 100 μl of a mixture of *Sfi *I-digested cDNA and xylene cyanol dye were applied to the matrix and allowed to fully absorb. One hundred microliters of column buffer were added to the matrix and allowed to fully absorb, then 600 μl of column buffer were added and single-drop fractions were collected in 16 tubes. The profile of the fractions was verified by running 3 μl of each fraction on a 1.1% agarose/EtBr gel at 150v for 10 minutes. The samples were then pooled into two size classes; fractions 7 and 8 were pooled into the "large size" and fractions 9 & 10 were pooled into the "small size".

From the libraries, 768 colonies were chosen for quality assurance tests. The bacterial transformants were amplified in selective 2xYT media, plasmids were purified by an alkaline lysis protocol according to the manufacturer's instructions (PerfectPrep, Eppendorf) and quantified by spectrophotometry. The molecular weights of cDNA inserts were measured by PCR amplification of cDNA inserts using the M13 vector primers M13fw (GTG TAA AAC GAC GGC CAG TAG) and M13rev (AAA CAG CTA TGA CCA TGT TCA C) followed by agarose gel electrophoresis against standards and visualized using a Kodak 440cf imaging station. Sequencing reactions were performed by priming at the 5' end of cDNA using vector primer pDNRlib30-50 (TAT ACG AAG TTA TCA GTC GAC G), ABI BigDye chemistry and the 3730 sequencer. Vector and poor quality sequences were trimmed from the sequencing reads and ESTs were assembled into contigs using the SeqManII software (DNASTAR package). Homologies with Genbank entries were discovered using Blastx against the non-redundant (nr) protein database. Those sequences with expectation-values better than 1 × 10^-27 ^were further examined for the presence of an annotated ATG start codon at the 5' end of the open reading frame (ORF). This last step was accomplished using NCBI's ORF finder tool [62]. Only those sequences whose Methionine aligned (including gaps) with the first amino acid of complete sequences were considered full-length transcripts.

### Characterization of the ESTs

On thousand twenty-eight additional cDNA samples were chosen for sequencing based on the microarray results obtained by Eads et al. (submitted) and Shaw et al. (submitted, and in prep). The sequencing reactions were carried out as outlined above. All 1,648 sequence reads from this study, with their quality scores, were obtained from ABI sequencer data files using phred [63] with default parameter values. The reads were then processed by discarding low quality and vector sequences using Lucy v1.19p [64] with default parameter values, by removing poly-A tails using EMBOSS trimest [65] and by discarding sequences with lengths under 100 bases. The remaining high quality EST set was reduced to a non-redundant set of unique gene transcripts by clustering with phrap [66] using the following parameters: mismatch penalty = -5; minimum match = 50; minimum score = 100. The resulting contigs and singlets that matched to mitochondrial gene transcripts (Genbank accession NC 000844) using Blastn were removed from subsequent analyses. To investigate whether the set of assembled sequences contain alternative transcripts of the same loci, the contigs and singlets were further clustered using the SeqManII software with the following relaxed parameters: match size = 12; maximum added gap length = 70; minimum percent match = 80; no gap penalty; gap length penalty = 0.70.

The putative open reading frames (ORFs) for the assembled sequences were determined in three steps using Prot4EST v2.2 [67] with DECODER having been disabled, ESTScan – which is an integral component of Prot4EST – and getorf from EMBOSS. The ORFs were selected during the first step when the assembled sequence translations aligned to proteins within the NCBI NR database with a Blastx e-value better than 1 × 10^-8^. Failing this first step, the ORFs where selected during the second step using ESTScan or simply by recording the longest uninterrupted ORFs when they were located on the positive stands of the sequences. Otherwise, the longest ORFs were selected during step three, based on the results obtained by using the EMBOSS program that restricted sequence translations from the negative strand. This restriction was justified by observing only 3 ORFs on the negative strand from among 376 predictions from step one.

Numerous sequence similarity searches were done for both the high quality EST set and the assembled sequences. First, queries were performed against the NCBI NR protein database (Genbank release 148) using a local installation of the WU-BLAST program [68]. The taxonomic domains were added to the results by parsing the taxa ID from the top match for each query and by retrieving the associated information from the NCBI [69]. Second, for a more confident assessment of whether the assembled sequences were shared with insects, they were compared to protein sequences archived in the NCBI UniGene sets for *Bombyx mori *(build #7), *Apis mellifera *(build #5), *Anopheles gambiae *(build #29) and *Drosophila melanogaster *(build #37) using tBlastx with an expectation threshold set at E < 0.005. The same search was performed against the *Caenorhabditis elegans *(build #23) UniGene database to judge whether the differences can be attributed to gains or losses within the representative insects or crustacean. The assembled sequences were clustered based on the distribution of bit scores across the databases using self organizing maps followed by k-means clustering within 28 nodes in Cluster v2.11 [70]. Third, to further ascertain whether the assembled sequences can be aligned to known proteins, queries were made against all *Drosophila melanogaster *gene translations that are predicted by the annotation v4.2.1 of the genome sequence assembly and against the genome nucleotide sequences of *Drosophila melanogaster *and *Caenorhabditis elegans *using tBlastx.

The assembled sequences were classified into gene ontology (GO)-defined functional classes using the program Blast2GO [22] and by extracting the GO annotations from FlyBase for sequences that strongly matched *D. melanogaster *gene transcripts. The putative gene annotations were examined for functional classes that are enriched within our lists of *Daphnia *genes compared to the total set of GO terms for all *Drosophila *genes using Gostat [71] and by testing for the enrichment of GO terms within subsets of the assembled sequences using Fisher's Exact Test executed within Blast2GO.

## Authors' contributions

JKC, BDE and JA conceived the study, designed and implemented the comparative analyses, and drafted the manuscript. JS and BDE performed the microarray experiments that guided the sequencing efforts, contributed the expression data and interpreted the results in light of this study. DB created the cDNA libraries. EB and JKC characterized the cDNA libraries and contributed EST sequences with BDE. All authors read and improved the final manuscript.

## Note added to proof

The recently released Draft *Daphnia pulex* genome   sequence (July 7, 2007) suggests that Daphnia possess a single copy of   the Ferritin 1 gene, represented by Singlet 73 on the phylogenetic tree   (Figure 3).  

## Supplementary Material

Additional file 1 Characterization of the *Daphnia pulex *EST sequences.
This file contains the following information about the analysis of EST sequences obtained for the study.
(1) EST information.
(2) Top match from Blastx searches of clustered *Daphnia *ESTs against the NCBI non-redundant (nr) protein database.
(3) Results using Blast2GO ().
(4) Top match from Blastx searches of clustered *Daphnia *ESTs against all *Drosophila melanogaster *predicted gene translations from annotation 4.2.1 – 16 columns describe the results. • Cluster id (*Daphnia*, this study). • Subject id (*D. melanogaster *dmel-all-translation-r4.2.1 data).
(5) Differential expression results in 3 microarray experiments.
(6) Top match from Blastx searches of *Daphnia *ESTs against NCBI non-redundant (nr) protein database – 12 columns describe the results. • Subject id of the best match in the nr database. Click here for file

Additional file 2 Supplemental Table 1. *Daphnia *genes annotated as regulators of transcription and translation based on sequence conservation with *Drosophila *genes with known functions. Scores are reported from results obtained by Blastx against all predicted translations from version 4.2.1 of the *D. melanogaster *genome annotation. First and second columns under DE show genes that are differentially expressed (+ = yes) in microarray experiments comparing male versus female transcripts and metals versus no metals exposure, respectively. TF = transcription factor; TR = transcriptional regulation; TE = transcript elongation; E = translation elongation; R = translation regulation. Click here for file

Additional file 3 Supplemental Table 2. *Daphnia *genes annotated as signaling proteins and other regulators based on sequence conservation with *Drosophila *genes with known functions. Scores are reported from results obtained by Blastx against all predicted translations from version 4.2.1 of the *D. melanogaster *genome annotation. First and second columns under DE show genes that are differentially expressed (+ = yes) in microarray experiments comparing male versus female transcripts and metals versus no metals exposure, respectively. Click here for file

Additional file 4 Supplemental Table 4. *Daphnia *genes annotated as candidates for gametogenesis based on sequence conservation with *Drosophila *genes with known functions. Processes include: SP = spermatogenesis; OO = oogenesis; FCD = follicle cell development; GCD = germ cell development; GT = gametogenesis. Two assembled sequences matched CG4027 and two other sequences matched CG2168. Click here for file

Additional file 5 Supplemental Table 4. *Daphnia *genes annotated as genes associated with exoskeletal function and molting. These include: CP = structural cuticle proteins; PM = peritrophic membrane; CM = cuticle metabolism; CA = chitinase; M = molting; CB = cuticle binding. Assignments are to proteins based on sequence conservation with *Drosophila *genes with known functions. Click here for file
