# Supplementary material for: Sampling Daphnia's expressed genes: preservation, expansion and invention of crustacean genes with reference to insect genomes
Source: BMC Genomics. 2007 Jul 6;8:217. doi: 10.1186/1471-2164-8-217 (PMC1940262; doi:10.1186/1471-2164-8-217)
Supplement: Additional file 2 — Supplemental Table 1. Daphnia genes annotated as regulators of transcription and translation based on sequence conservation with Drosophila genes with known functions. Scores are reported from results obtained by Blastx against all predicted translations from version 4.2.1 of the D. melanogaster genome annotation. First and second columns under DE show genes that are differentially expressed (+ = yes) in microarray experiments comparing male versus female transcripts and metals versus no metals exposure, respectively. TF = transcription factor; TR = transcriptional regulation; TE = transcript elongation; E = translation elongation; R = translation regulation. [file 1471-2164-8-217-S2.pdf]

Suppl. Table 1. *Daphnia* genes annotated as regulators of transcription and translation based on sequence conservation with *Drosophila* genes with known functions. Scores are reported from results obtained by Blastx against all predicted translations from version 4.2.1 of the *D. melanogaster* genome annotation. First and second columns under DE show genes that are differentially expressed (+ = yes) in microarray experiments comparing male versus female transcripts and metals versus no metals exposure, respectively. TF=transcription factor; TR=transcriptional regulation; TE=transcript elongation; E=translation elongation; R=translation regulation.

Daphnia ID	Function	*Drosophila* gene name	FlyBase ID	% Similarity	E-value	Bit score	DE
Transcriptional Regulation							
Singlet 339	TF/kinase	*Cyclin Dependent kinase 7*	FBgn0015617	79	1E-101	365	-, -
Singlet 269	GTPase	*Rab-protein 11*	FBgn0015790	82	8E-96	346	-, -
Singlet 163	Chromatin binding	*Dorsal Switch protein 1*	FBgn0011764	75	3E-72	268	+, -
Contig 47	Histone binding	*Nucleosome Assembly protein 1*	FBgn0015268	48	2E-56	216	+, -
Contig 211	TR	*Ribosomal protein S27A*	FBgn0003942	91	8E-56	213	+, -
Singlet 182	TE	*Elongin-C*	FBgn0023211	88.	3E-55	211	+, -
Singlet 379	TF/pyruvate metab	*CG11876*	FBgn0039635	80	6E-52	198	-, -
Contig 52	TR	*Cysteine Proteinase 1*	FBgn0013770	55	1E-50	196	+, +
Singlet 21	TR	*enhancer of rudimentary*	FBgn0011586	84	2E-47	184	+, +
Singlet 191	TF	*maf-S*	FBgn0034534	66	1E-40	162	+, -
Contig 153	TR	*Ribosomal Protein L4*	FBgn0003941	62	2E-36	147	+, -
Singlet 418	CG3224	*CG3224*	FBgn0029885	50	5E-34	140	-, +
Singlet 235	TF	*Meiotic central spindle*	FBgn0025874	38	1E-32	136	+, -
Singlet 225	TR/Wnt signaling	*Shaggy*	FBgn0003371	67	1E-21	100	-, -
Singlet 495	TR	*CG18619*	FBgn0032202	66	2E-17	85	-, +
Singlet 469	TR	*Similar to Deadpan*	FBgn0032741	36	6E-17	84	-, +
Singlet 215	TF	*Ftz transcription factor f1*	FBgn0001078	79	4E-16	81	-, -
Translational Regulation							
Contig 238	mRNA binding	*Ribosomal protein LP0*	FBgn0000100	71	1E-119	426	+, +
Contig 258	E	*Elongation factor 1alpha100E*	FBgn0000557	92	1E-103	373	+, +
Contig 224	R	*Ribosomal protein S6*	FBgn0004922	72	4E-86	314	+, -
Singlet 406	E	*Elongation factor 2b*	FBgn0000559	89	1E-82	302	+, -
Singlet 138	R	*Initiation Factor-5A*	FBgn0034967	79	4E-71	264	+, -
Singlet 362	E	*Elongation factor 1 gamma*	FBgn0029176	57	1E-65	246	+, -
Contig 194	E	*Elongation factor 1beta*	FBgn0028737	59	4E-57	218	+, -
Singlet 136	Negative R	*CG15261*	FBgn0028510	48	2E-32	135	+, -
Singlet 187	CG9099	*CG9099*	FBgn0030802	54	4E-29	124	+, -
Singlet 447	E	*Ribosomal protein LP1*	FBgn0002593	71	4E-26	114	+, -
Singlet 239	R	*Thor*	FBgn0022073	40	4E-19	92	-, -
Contig 265	E	*Ribosomal protein LP2*	FBgn0003274	61	5E-12	67	+, +
Singlet 19	R	*piwi*	FBgn0004872	32	4E-12	66	-, +
Regulation of Transcription and Translation							
Contig 76	TR, R	*bicoid*	FBgn0000181	60	9E-41	163	+, -
Contig 136	TR, R	*Ribosomal protein L7*	FBgn0005593	67	3E-78	288	+, -
