# Supplementary material for: Sampling Daphnia's expressed genes: preservation, expansion and invention of crustacean genes with reference to insect genomes
Source: BMC Genomics. 2007 Jul 6;8:217. doi: 10.1186/1471-2164-8-217 (PMC1940262; doi:10.1186/1471-2164-8-217)
Supplement: Additional file 3 — Supplemental Table 2. Daphnia genes annotated as signaling proteins and other regulators based on sequence conservation with Drosophila genes with known functions. Scores are reported from results obtained by Blastx against all predicted translations from version 4.2.1 of the D. melanogaster genome annotation. First and second columns under DE show genes that are differentially expressed (+ = yes) in microarray experiments comparing male versus female transcripts and metals versus no metals exposure, respectively. [file 1471-2164-8-217-S3.pdf]

Suppl. Table 2. *Daphnia* genes annotated as signaling proteins and other regulators based on sequence conservation with *Drosophila* genes with known functions. Scores are reported from results obtained by Blastx against all predicted translations from version 4.2.1 of the *D. melanogaster* genome annotation. First and second columns under DE show genes that are differentially expressed (+ = yes) in microarray experiments comparing male versus female transcripts and metals versus no metals exposure, respectively.

Daphnia ID	Pathway	*Drosophila* gene name	FlyBase ID	% Similarity	E-value	Bit score	DE
Signaling Proteins							
Singlet 229	JNK/NOT GTPase	*Cdc42*	FBgn0010341	93	5E-102	367	-, -
Singlet 82	Notch	*O-fucosyl transferase 1*	FBgn0033901	63	2E-87	318	-, -
Singlet 35	Rhodopsin	*Calmodulin*	FBgn0000253	100	7E-81	296	-, -
Contig 115	PKC	*Rec act PkC1*	FBgn0020618	78	3E-80	294	+, -
Contig 213	Chitinase	*CG2989*	FBgn0030171	37	7E-73	271	+, +
Singlet 271	Osk localization	*Mago nashi*	FBgn0002736	86	6E-72	266	-, -
Singlet 151	Calmodulin	*CG2185*	FBgn0037358	70	1E-64	242	+, -
Singlet 463	GTP Reception	*Rheb*	FBgn0041191	62	1E-62	236	-, -
Singlet 373	JAK/STAT	*cdc2c*	FBgn0004107	63	3E-59	224	-, -
Singlet 371	Signal Recognition	*l(1)G0320*	FBgn0028327	60	1E-42	169	-, -
Singlet 367	GTPase	*Rab4*	FBgn0016701	62	1E-36	149	+, -
Contig 227	GTP Reception	*Rhodopsin 6*	FBgn0019940	39	1E-32	136	+, +
Singlet 43	Acetylcholine	*NicAcytRec α-96Ab*	FBgn0000039	31	2E-29	125	-, -
Contig 64	Receptor signaling	*CG32262*	FBgn0052262	40	1E-23	106	+, +
Singlet 197	Receptor binding	*CG3153*	FBgn0038198	36	6E-19	91	+, -
Singlet 486	GTPase	*Ggamma1*	FBgn0004921	61	2E-16	81	-, +
Contig 161	Receptor binding	*NPC2*	FBgn0031381	33	2E-15	79	+, +
Singlet 184	Chitinase	*Chitinase 4*	FBgn0022700	51	8E-15	77	+, -
Singlet 147	Kinase	*CG15072*	FBgn0034376	28	7E-13	71	+, +
Contig 186	Endopeptidase	*Papilin*	FBgn0003137	48	7E-09	57	+, +
Singlet 37	Chitinase	*Chitinase 4*	FBgn0022700	47	3E-08	53	-, +
Singlet 348	GTP/Photo Reception	*Rhodopsin 2*	FBgn0003248	52	6E-07	50	+, +
Other Regulatory Proteins							
Singlet 299	Caspase/apoptosis	*Ice*	FBgn0019972	44	8E-54	206	+, -
Singlet 281	GTPase/molting	*bursicon*	FBgn0038901	71	6E-49	190	+, -
Singlet 404	Actin bind/cell adhesion	*Mp20*	FBgn0002789	58	2E-42	168	+, -
Singlet 334	Osk localization	*tsunagi*	FBgn0033378	75	2E-37	151	-, -
Singlet 280	Regulation of Proteolysis	*Nedd8*	FBgn0032725	94	6E-26	112	-, -
Singlet 294	Nucleosome assembly	*His3.3A*	FBgn0014857	84	1E-22	101	+, -
