# Supplementary material for: Sampling Daphnia's expressed genes: preservation, expansion and invention of crustacean genes with reference to insect genomes
Source: BMC Genomics. 2007 Jul 6;8:217. doi: 10.1186/1471-2164-8-217 (PMC1940262; doi:10.1186/1471-2164-8-217)
Supplement: Additional file 4 — Supplemental Table 4. Daphnia genes annotated as candidates for gametogenesis based on sequence conservation with Drosophila genes with known functions. Processes include: SP = spermatogenesis; OO = oogenesis; FCD = follicle cell development; GCD = germ cell development; GT = gametogenesis. Two assembled sequences matched CG4027 and two other sequences matched CG2168. [file 1471-2164-8-217-S4.pdf]

Suppl. Table 3. *Daphnia* genes annotated as candidates for gametogenesis based on sequence conservation with *Drosophila* genes with known functions. Processes include: SP=spermatogenesis; OO=oogenesis; FCD=follicle cell development; GCD=germ cell development; GT=gametogenesis. Two assembled sequences matched CG4027 and two other sequences matched CG2168.

Daphnia ID	Pathway	*Drosophila* gene name	FlyBase ID	% Similarity	E-value	Bit score
Contig 31	*SP*	*Actin5C*	FBgn0000042	87	3E-103	370
Singlet 229	*OO, FCD*	*Cdc42*	FBgn0010341	93	5.00E-102	367
Singlet 269	*OO, GCD*	*Rab11*	FBgn0015790	82	8.00E-96	346
Contig 67	*OO*	*Ribosomal protein S3A*	FBgn0017545	72	1.00E-88	322
Singlet 271	*OO,GCD*	*Mago nashi*	FBgn0002736	86	6.00E-72	266
Singlet 445	*OO*	*Sans fille*	FBgn0003449	69	4.00E-68	254
Singlet 361	*GCD*	*Tropomyosin1*	FBgn0003721	77	9.00E-64	239
Contig 25	*SP*	*Actin 5C*	FBgn0000042	98	1E-46	182
Contig 76	*OO*	*bicaudal*	FBgn0000181	60	9.00E-41	163
Singlet 235	*GT*	*Meiotic central spindle*	FBgn0025874	38	1E-32	136
Singlet 468	*SP,OO,GCD*	*chickadee*	FBgn0000308	58	2.00E-38	155
Singlet 334	*OO,GCD*	*tsunagi*	FBgn0033378	75	2.00E-37	151
Contig 27	*OO*	*RpS3A*	FBgn0017545	64	3.00E-36	148
Singlet 125	*OO*	*twinstar*	FBgn0011726	38	8.00E-28	120
Singlet 447		*Ribosomal protein LP1*	FBgn0002593	71	4E-26	114
Singlet 225	*OO*	*shaggy*	FBgn0003371	67	1.00E-21	100
Singlet 284	*OO*	*Suppressor of Profilin*	FBgn0001961	66	4.00E-08	52.8
