# Supplementary material for: Sampling Daphnia's expressed genes: preservation, expansion and invention of crustacean genes with reference to insect genomes
Source: BMC Genomics. 2007 Jul 6;8:217. doi: 10.1186/1471-2164-8-217 (PMC1940262; doi:10.1186/1471-2164-8-217)
Supplement: Additional file 5 — Supplemental Table 4. Daphnia genes annotated as genes associated with exoskeletal function and molting. These include: CP = structural cuticle proteins; PM = peritrophic membrane; CM = cuticle metabolism; CA = chitinase; M = molting; CB = cuticle binding. Assignments are to proteins based on sequence conservation with Drosophila genes with known functions. [file 1471-2164-8-217-S5.pdf]

Suppl. Table 4. *Daphnia* genes annotated as genes associated with exoskeletal function and molting. These include: CP=structural cuticle proteins; PM=peritrophic membrane; CM=cuticle metabolism; CA=chitinase; M=molting; CB=cuticle binding. Assignments are to proteins based on sequence conservation with *Drosophila* genes with known functions.

Daphnia ID	Pathway	*Drosophila* gene name	FlyBase ID	% Similarity	E-value	Bit score
Contig 20	*CP*	*CG6305*	FBgn0033869	57	3.00E-15	78.2
Contig 180	*CP*	*CG6305*	FBgn0033869	58	7.00E-14	73.9
Contig 19	*CP*	*CG6305*	FBgn0033869	53	2.00E-16	82.8
Contig 24	*CP*	*CG6305*	FBgn0033869	40	3.00E-16	82
Contig 23	*CP*	*CG6305*	FBgn0033869	50	1.00E-11	67
Contig 241	*CP*	*CG6305*	FBgn0033869	50	9.00E-12	67
Contig 257	*CP*	*CG6305*	FBgn0033869	49	7.00E-11	64.3
Singlet 158	*CP*	*CG6305*	FBgn0033869	61	3.00E-19	91.7
Singlet 262	*CP*	*CG6305*	FBgn0033869	69	3.00E-08	53.1
Singlet 433	*CP*	*CG6305*	FBgn0033869	63	2.00E-16	82.4
Singlet 516	*CP*	*CG6305*	FBgn0033869	58	8.00E-09	55.1
Singlet 388	*CP*	*CG6305*	FBgn0033869	57	3.00E-17	85.1
Contig 120	*CP*	*CG6305*	FBgn0033869	51	7.00E-16	79.7
Contig 149	*CP*	*CG6305*	FBgn0033869	59	1.00E-09	60.5
Contig 182	*CP*	*CG6305*	FBgn0033869	55	6.00E-09	55.5
Singlet 416	*CP*	*CG7658*	FBgn0037069	55	3.00E-08	53.1
Contig 15	*CP*	*CG7658*	FBgn0037069	61	4.00E-09	56.2
Contig 244	*CP*	*CG7658*	FBgn0037069	61	4.00E-09	58.2
Contig 66	*CP*	*CG7658*	FBgn0037069	61	2.00E-08	56.2
Singlet 448	*CP*	*CG8511*	FBgn0033730	41	3.00E-10	61.6
Contig 166	*CP*	*CG8511*	FBgn0033730	39	4.00E-09	58.2
Contig 70	*CP*	*CG8511*	FBgn0033730	38	1.00E-08	56.6
Singlet 57	*PM,CM*	*CG6947*	FBgn0036233	28	2.00E-09	59.3
Singlet 316	*PM, CM*	*CG6947*	FBgn0036233	22	6.00E-09	57.8
PSinglet 424	*PM, CM*	*CG6947*	FBgn0036233	26	4.00E-08	54.7
Singlet 251	*CA*	*CG9307*	FBgn0038180	37	8.00E-35	143
Singlet 498	*CA*	*CG9307*	FBgn0038180	40	4.00E-05	42.7
Contig 218	*CA*	*CG9307*	FBgn0038180	38	8.00E-29	124
Singlet 302	*AD*	*Salivary gland secretion1*	FBgn0003372	34	0.001	40
Singlet 450	*AD*	*Sgs1*	FBgn0003372	38	3.00E-04	41.6
Singlet 184	*CA*	*Chitinase4*	FBgn0022700	51	8.00E-15	77
Singlet 37	*CA*	*Chitinase4*	FBgn0022700	47	3.00E-08	53.1
Singlet 223	*CP*	*CG8505*	FBgn0033728	48	1.00E-22	102
Contig 32	*CP*	*CG8505*	FBgn0033728	51	2.00E-23	105
Singlet 493	*CP*	*CG15920*	FBgn0034157	48	8.00E-11	63.2
Contig 49	*CP*	*CG15920*	FBgn0034157	53	1.00E-15	79.3
Singlet 470	*CP*	*CG12330*	FBgn0035686	35	1.00E-08	57
Contig 132	*CP*	*CG12330*	FBgn0035686	35	1.00E-08	55.5
Singlet 295	*PM*	*CG10154*	FBgn0036361	43	1.00E-09	58.5
Contig 77	*PM*	*CG10154*	FBgn0036361	41	3.00E-08	54.3
Singlet 215	*M*	*ftz-f1*	FBgn0001078	46	4.00E-16	80.9
Singlet 506	*PM, CM*	*CG4778*	FBgn0027600	51	2.00E-53	205
Contig 213	*CA, CM*	*CG2989*	FBgn0030171	37	7.00E-73	271
Singlet 322	*PM, CM*	*CG11142*	FBgn0031737	43	1.00E-44	176
Singlet 165	*PM, CM*	*CG1869*	FBgn0035398	40	5.00E-17	83.6

Singlet 314	*CB, CM*	*CG12009*	FBgn0035430	61	1.00E-31	132
Singlet 2	*CP*	*CG3672*	FBgn0035985	40	3.00E-07	52
Contig 209	*CP*	*CG4818*	FBgn0036617	40	4.00E-10	62.4
Contig 273	*CP*	*CG6240*	FBgn0038714	31	9.00E-04	40.8
Singlet 281	*Molting*	*bursicon*	FBgn0038901	71	6.00E-49	190
Singlet 308	*CP*	*CG15884*	FBgn0039481	38	9.00E-20	94
Singlet 161	*CB, CM*	*CG31973*	FBgn0051973	45	6.00E-14	73.2
Contig 173	*CB, CM*	*CG32209*	FBgn0052209	38	4.00E-34	141
